# Vector Bundle Model of Complex Electromagnetic Space and Change Detection

**DOI:** 10.3390/e21010010

**Published:** 2018-12-23

**Authors:** Hao Wu, Yongqiang Cheng, Xiaoqiang Hua, Hongqiang Wang

**Affiliations:** College of Electronic Science, National University of Defense Technology, Changsha 410073, China

**Keywords:** complex electromagnetic space, vector bundle, change detection, information geometry, geometric detector

## Abstract

Complex Electromagnetic Space (CEMS), which consists of physical space and the complex electromagnetic environment, plays an essential role in our daily life for supporting remote communication, wireless network, wide-range broadcast, etc. In CEMS, the electromagnetic activities might work differently from the ideal situation; the typical case is that undesired signal would disturb the echo of objects and overlap into it resulting in the mismatch of matched filter and the reduction of the probability of detection. The lacking mathematical description of CEMS resulting from the complexity of electromagnetic environment leads to the inappropriate design of detection method. Therefore, a mathematical model of CEMS is desired for integrating the electromagnetic signal in CEMS as a whole and considering the issues in CEMS accurately. This paper puts forward a geometric model of CEMS based on vector bundle, which is an abstract concept in differential geometry and proposes a geometric detector for change detection in CEMS under the geometric model. In the simulation, the proposed geometric detector was compared with energy detector and matched filter in two scenes: passive detection case and active detection case. The results show the proposed geometric detector is better than both energy detector and matched filter with 4∼5 dB improvements of SNR (signal-to-noise ratio) in two scenes.

## 1. Introduction

Complex electromagnetic space (CEMS), which consists of physical space and the complex electromagnetic environment, plays an important role in our daily life for supporting remote communication, wireless network, wide-range broadcast, etc. The complex electromagnetic environment consists of plentiful varieties of radio waves from extensive sensors dynamically overlapping in the time, frequency and power domain. Because of the complex electromagnetic environment, electromagnetic activities, such as our daily communication, military mission and disaster rescue, might be disturbed and unable to work normally. The typical case is that the matched filter detection method would fail to discover the target resulting from the mismatch of received signal, which contains many signals from other unknown radiation sources. Therefore, it is necessary and important to study CEMS for building an appropriate model to express the electromagnetic signal and propose a novel method for change detection, which is a basic issue but might be different in CEMS.

Electromagnetic signal in CEMS contains natural and artificial, deterministic and stochastic, and antagonistic and non-antagonistic signals. The complexity of electromagnetic signal results in the difficulty of describing the complex electromagnetic environment. There are many significant works concerning the complex electromagnetic environment, achieving some pioneering research. The ECCM (electronic counter-countermeasures) combat model is proposed to suit the needs of EW (electronic warfare) combat in [1]. The mathematical models of radio frequency noise jamming and range-gate pull off is built in [2,3]. In [4], a method of calculating the complexity of CEMS is proposed and the types of CEMS are listed according to the signals, radiation sources, etc. However, current research does not provide a global and feasible model of CEMS to support expected works, such as change detection and object sensing.

Actually, in the real work, the observed electromagnetic signal is not deterministic but stochastic, resulting from the noise deriving from receiver or measurement. Moreover, the complexity and uncertainty of electromagnetic signal resulting from the overlapping of sorts of signal and noise cause the demand of probability distribution to describe the electromagnetic signal of each point in CEMS. In current research concerning statistics inference, especially statistical signal processing, the statistical issues are often treated in a geometrical view, in which a family of probability distributions is regarded as a statistical manifold [5].

In CEMS, the probability distribution of each point could be parameterized as a vector, which is also the coordinate of this distribution on the statistical manifold. The vectors over all points in CEMS build up a vector field, which is described as a section of vector bundle in geometric view. Vector bundle is a topological construction in differential geometry, which denotes a vector space family parameterized by a manifold [6], and many fundamental concepts are the special cases of vector bundle, such as electromagnetic field, gravitational field, energy field and other space-like concepts. This paper combines the vector bundle and statistical manifold to build a statistical model of CEMS that provides a global model to analyze the issues of CEMS such as change detection.

Moreover, change detection is one of the most critical issues in CEMS and is different from traditional detection modes. One of the traditional modes is that radar emits a signal and receives echos for detection [7,8,9]; this mode is effective but easily disturbed. Furthermore, passive mode has advantage in terms of hiding, and has also been studied [10,11,12,13]. Passive mode utilizes signal of radiation source, such as the communication signal, for detection. In CEMS, the knowledge concerning the environment is often absent; the detection only depends on the collection of data. Therefore, it is a data-driven detection mode in CEMS. Information geometry has the capacity of solving the problems in CEMS because there are many achievements in statistical signal processing, especially detection problem, based on information geometry. Information geometry was pioneered by Rao [14]; further developed by Chentsov [15], Efron [16,17] and Amari [18,19]; and has found a wide range of applications [20,21,22,23,24]. In detection theory, Barbaresco et al. [25,26] are the pioneers and creatively proposed a matrix CFAR based on Riemannian distance, which is a major breakthrough and has better detection performance than classical CFAR detector. Based on these significant works, in our previous studies [20,27,28,29,30], the geometric detector introduced from the matrix manifold utilizing information divergence instead of Riemannian distance also achieves good performance and requires little computation.

In this paper, the signal in CEMS is treated as a probability distribution of the complexity and uncertainty. For considering the electromagnetic signal as an entirety, the mathematical model of CEMS is proposed based on vector bundle and statistical manifold in this paper. In this model, a specific CEMS corresponds to a section of the proposed vector bundle, so the change of CEMS corresponds to the difference of sections. The difference between two sections is modeled as a function based on Riemannian distance of statistical manifold and then the function is quantified as a real number using the $L_{k}$ norm. In addition, the quantified difference is proven to be a distance of section that satisfies positive definiteness, symmetry and triangle inequality. The proposed detector of change detection in this paper utilizes the distance of section as the test statistics. In the simulation, we compared the detection performance of geometric detector with energy detector and matched filter in two scenes: passive detection case and active detection case. The results show geometric detector is better than both energy detector and matched filter with 3∼4 dB improvement of SNR (signal-to-noise ratio) in the two scenes.

The rest of the paper is organized as follows. In Section 2, the model of CEMS based on vector bundle is introduced. In Section 3, the definition of distance of section is presented and the geometric detection method based on the distance follows. Then, the related simulation scenes and the results are presented in Section 4. Finally, the concise conclusion of the paper is provided in Section 5.

## 2. Vector Bundle Model

### 2.1. The Signal Model in CEMS

Complex electromagnetic space (CEMS) consists of a physical space, in which each point is covered by electromagnetic signals. This processing can also be modeled mathematically, i.e., CEMS is a mapping that maps the points in the physical space to electromagnetic signals in signal space.

**Figure 1:**
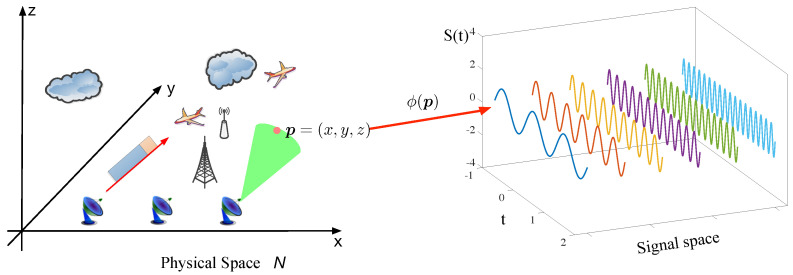
The map model of complex electromagnetic space.

In Figure 1, the mapping model of CEMS is illustrated. The mapping ϕ(p) indicates the CEMS, in which the point p=(x,y,z) is mapped to the signal marked $S_{p}\left(t\right)$ that is one of the elements in the signal space. The form of electromagnetic signal is determined by the complex electromagnetic environment and considers, e.g., the types and position of radiation source, reflection and other related things, so it is sophisticated and unknown in CEMS. In fact, the electromagnetic signal we obtained is often with noise, so we just directly consider the electromagnetic signal as observed signal $I_{p}\left(t\right)$, which consists of $S_{p}\left(t\right)$ and noise as Equation (1),

(1) $$I_{p}\left(t\right)=S_{p}\left(t\right)+w_{p}\left(t\right)$$

where $w_{p}\left(t\right)$ is often white Gaussian noise (WGN). Because of the complexity and uncertainty of electromagnetic signal resulting from the overlapping of sorts of signal and noise, the signal is desired to be treated in statistical way, i.e., the signal is modeled as a probability distribution. With this connection, the signal space also can be regarded as a statistical manifold, which consists of a parameterized family of distribution,

(2) $$M=\{p_{x}\left(x;\xi \right)|\xi \in R^{m}\}$$

where *M* is called a *m*-dimension manifold.

**Figure 2:**
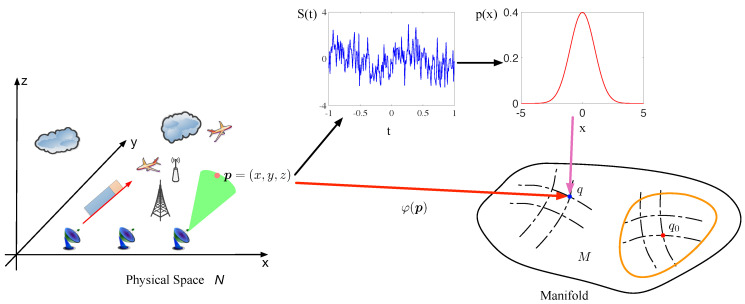
The process of signal geometric model building.

As shown in Figure 2, the electromagnetic signal on point p=(x,y,z) corresponds to a point on the statistical manifold. Mapping the electromagnetic signals onto the manifold is convenient for comparing the difference between two signals in statistical view, by calculating the distance between two corresponding points on the manifold. That means the change of the electromagnetic signals can be quantified as the distance on the manifold. Naturally, this concept could help us conceive a novel method to detect the change of electromagnetic environment.

### 2.2. The Vector Bundle Model of CEMS

After modeling the signal as a point on the manifold, CEMS is similar to a field, which is a basic concept in physics and similarly related to a vector bundle on geometric view.

In differential geometry, a vector bundle is defined as a triple (E,N,π), which *E* is called the bundle space, *N* is called the base space and π is called the bundle projection, which maps the elements from *E* to *N*, i.e., π:E→N. Furthermore, the projection π should satisfy that $\forall p\in N,\pi^{-1}\left(p\right)$ is differential homeomorphic to $R^{m}$. Then, the (E,N,π) is called a (real) *m*-dimensional vector bundle [31].

The vector bundle model of CEMS is formulated as (E,N,π), in which base space *N* indicates the physical space, bundle space E=N×M is a product space of *N* and statistical manifold *M* and bundle projection π maps a pair (p,q) from N×M to p from *N*, i.e., π:N×M→N and π:(p,q)↦p. For convenience, in the following, another projection $\pi_{M}:\;N\times M\to M$ is defined as

(3) $$\pi_{M}:\;\left(p,q\right)\mapsto q.$$

Then, naturally, the preimage set $\pi^{-1}\left(p\right)=\left\{p\right\}\times M$ is homeomorphic to the statistical manifold *M* with projection $\pi_{M}$. Because the statistical manifold that consists of a family of distributions often has the global coordinate chart, the $\pi^{-1}\left(p\right)$ is also differential homeomorphic to $R^{m}$. Thus, the triple (E,N,π) satisfies the definition of vector bundle.

**Figure 3:**
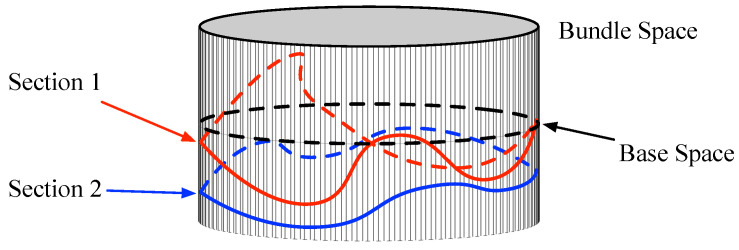
The section in the vector bundle.

However, the bundle space is not the model of CEMS; the meaning of bundle space is a higher level to CEMS, where the CEMS is similar to a subset of bundle space, and even all possible statuses of the CEMS correspond to a subset of the bundle space. In fact, the section of vector bundle corresponds to the CEMS, which is a map s:N→E that satisfies π∘s=id:N→N. As illustrated in Figure 3, the vector bundle also can be treated as a surface, and the section is a curve that can be projected to the full base space. As shown in Figure 2, the electromagnetic signal of each point in the physical space is mapped to a point on the statistical manifold, which is similar to the section of vector bundle model and the φ(p) in Figure 2 can be connected to section s(p) with $\pi_{M}$ as $\varphi \left(p\right)=\pi_{M}\left(s\left(p\right)\right)$.

In general, because of the complexity and uncertainty of the electromagnetic signal, the signal in CEMS is described in a statistical way. Thus, the electromagnetic signal of each point is described as a vector that indicates a statistical parameter of a distribution and corresponds to a point on the statistical manifold. The main idea of the proposed model is integrating these vector as a section of vector bundle to describe the CEMS.

## 3. Change Detection

### 3.1. Framework of Detection Method

In CEMS, the change often means the presence of a target, the moving of object or other emergencies and always results in the varying of electromagnetic signal. Meanwhile, in the vector bundle model of CEMS, the section has also changed following the change of CEMS. Then, the problem can be formulated as

(4) $$\begin{array}{c} H_{0}:\;s=s_{0}\\ H_{1}:\;s\neq s_{0} \end{array}$$

where *s* means the section of current CEMS and $s_{0}$ means the section of initial CEMS.

**Figure 4:**
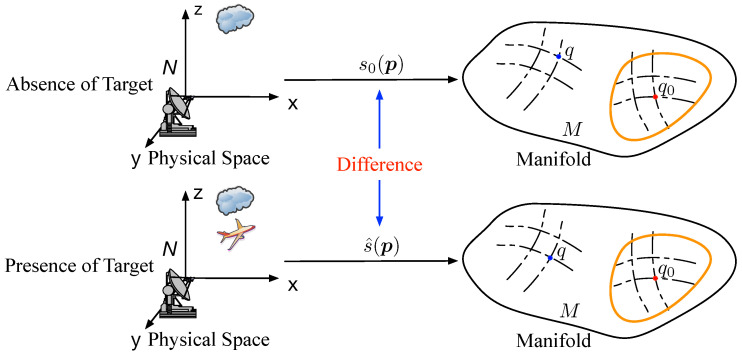
The change of section by the presence of object.

Therefore, it is possible to propose a method to detect the change of CEMS by comparing the difference of two sections, as illustrated in Figure 4.

Based on this idea, the framework of detection method consists of the following steps:

1. Model the initial CEMS as section $s_{0}$.
2. Obtain the electromagnetic signals and estimate the probability distributions of them.
3. According to the estimated distribution, get the estimated section $\hat{s}$.
4. Judge the difference between $s_{0}$ and $\hat{s}$.

However, the difference of section is not clear, because there is no common definition about that. To solve this problem, reviewing the important notions in information geometry is significant.

### 3.2. Distance on Statistical Manifold

On statistical manifold, the Riemannian metric is defined as Fisher information matrix G(ξ) [32]

(5) $${\left[G\left(\xi \right)\right]}_{ij}=g_{ij}\left(\xi \right)=-E\left[\frac{\partial^{2}\ln p(x;\xi )}{\partial \xi^{i}\partial \xi^{j}}\right].$$

In Riemannian manifold, the length of curve γ(t), $t_{0}\leq t\leq t_{1}$ is defined with the Riemannian metric as [31]

(6) $$∥\gamma \left(t\right)∥=\int_{t_{0}}^{t_{1}}\sqrt{\sum_{i,j}g_{ij}\left(\gamma \left(t\right)\right)\frac{d\gamma^{i}}{dt}\frac{d\gamma^{j}}{dt}}dt.$$

The distance between two distributions $p(x;\xi_{1})$ and $p(x;\xi_{2})$ is the minimum length over all possible curves connecting the two points on the manifold [33]; it is named geodesic distance. However, it is not easy to calculate the distance between two points on the manifold; an integration should be calculated and differential equations should be solved first [31]. Fortunately, in local area, the geodesic distance and Kullback–Leibler (KL) divergence satisfy [5,34],

(7) $$2KL\left(p\left(x;\xi \right)∥p\left(x;\xi +d\xi \right)\right)=\sum_{i,j}g_{ij}\left(\xi \right)d\xi^{i}d\xi^{j}.$$

Therefore, Equation (7) provides another method to calculate the distance between two neighboring points, by calculating the KL divergence [35]. Furthermore, KL divergence is defined as

(8) $$KL\left(p\left(x;\xi_{1}\right)∥p\left(x;\xi_{2}\right)\right)=\int p\left(x;\xi_{1}\right)\ln\frac{p(x;\xi_{1})}{p(x;\xi_{2})}\;dx,$$

which can be expressed as a closed form in general situations, such as normal distribution.

### 3.3. Metric of Section

The section *s* is a mapping from physical space *N* to bundle space E=N×M and satisfies s(p)=(p,q). Therefore, for different sections, the images of point p are the same in the first element. The difference among the images of point p depends on the second element, which is a point on the statistical manifold. The distance on the statistical manifold, which is introduced in Section 3.2, can be used for quantifying the difference among the images of same point under different sections. Actually, the difference between two sections $s_{1}$ and $s_{2}$ consists of the differences between the images $s_{1}\left(p\right)$ and $s_{2}\left(p\right)$ over all points p in the physical space.

**Figure 5:**
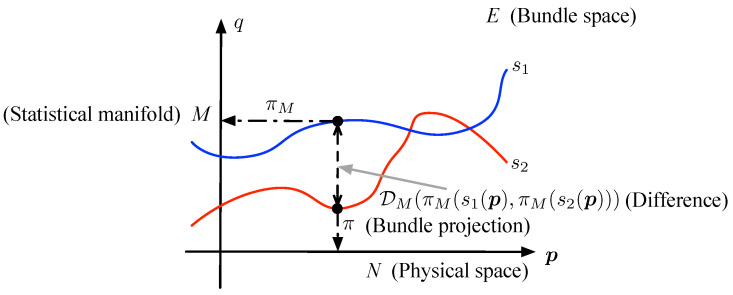
The difference between two sections.

Thus, as shown in Figure 5, we can introduce Equation (9) to consider the difference,

(9) $$\Gamma_{s_{1},s_{2}}\left(p\right)=D_{M}\left(\pi_{M}\left(s_{1}\left(p\right)\right),\pi_{M}\left(s_{2}\left(p\right)\right)\right),$$

where $D_{M}\left(q_{1},q_{2}\right)$ means the distance between $q_{1}$ and $q_{2}$ on statistical manifold, $\pi_{M}$ is introduced in Section 2.2 and means the projection from N×M to *M*. This function means the distance between images of the same point under two sections $s_{1}$ and $s_{2}$, and is often continuous, because the electromagnetic signal is continuous in physical space. Furthermore, it is reasonable to quantify the difference of sections based on the function, because there are many ways to define the distance of function [36], such as the norm of function. Therefore, we can quantify the distance between $s_{1}$ and $s_{2}$ as the norm of function $\Gamma_{s_{1},s_{2}}$,

(10) $$D_{s}\left(s_{1},s_{2}\right)=∥\Gamma_{s_{1},s_{2}}∥.$$

The norm of function can introduce the distance and satisfies the positive definiteness, homogeneity and triangle inequality. The widely used norm is $L_{k}$-norm, which is [37]

(11) $$∥f{∥}_{k}=(\int_{D(f)}{\left|\;f\left(x\right)\;\right|}^{k}{\;dx)}^{\frac{1}{k}},$$

where *k* is a real number (often integer) that is no less than 1 and D(f) means the domain of definition of *f*. Especially, $L_{\infty }$-norm is [37]

(12) $$∥f{∥}_{\infty }=\underset{D(f)}{\sup}\left|f(x)\right|$$

where $\sup_{D(f)}f(x)$ means the supremum of function *f* over D(f).

Actually, when the norm in Equation (10) is $L_{k}$-norm, we have the following theorem.

**Theorem** **1.** *$D_{s}$ satisfies the positive definiteness, symmetry and triangle inequality, if the norm of function is $L_{k}$-norm.*

The detailed proof of Theorem 1 is in Appendix A. Although the importance of Theorem 1 is limited in the geometric detector, it is also with definite theoretical meaning.

As Theorem 1, $D_{s}$ is actual distance of sections. Based on that, the rule of detection is formulated as

(13) $$D_{s}\left(\hat{s},s_{0}\right)\underset{H_{0}}{\overset{H_{1}}{≷}}\eta ,$$

where η is the threshold, $\hat{s}$ is the estimated section with observed signal and $s_{0}$ is the initial section.

To sum up, the main idea of the proposed detection method is combining the quantified difference in probability distribution of each point between $H_{1}$ and $H_{0}$ with the norm of function and utilizing it as test statistic to judge whether the target is present or absent.

### 3.4. The Section of CEMS in Real Work

In fact, getting the section of CEMS might be impossible in our real work, because the point is continuous in the physical space; it is infinite. Thus, one of the available methods is sampling the physical space as finite points. Meanwhile, changing the $L_{k}$-norm to $l_{k}$-norm, which are applied in function and sequence, respectively, the distance of section is formulated as

(14) $$D_{s}\left(s_{1},s_{2}\right)=(\sum_{i=1}^{n}|\;\Gamma_{s_{1},s_{2}}\left(p_{i}\right)\;{{|}^{k})}^{\frac{1}{k}},$$

where $p_{k}$ is the *k*th sampling point and *n* is the number of sampling points. Furthermore, the $l_{\infty }$-norm would introduce

(15) $$D_{s}\left(s_{1},s_{2}\right)=\max_{i=1,\ldots ,n}|\;\Gamma_{s_{1},s_{2}}\left(p_{i}\right)\;|.$$

## 4. Simulation

### 4.1. Simulation Scene

**Figure 6:**
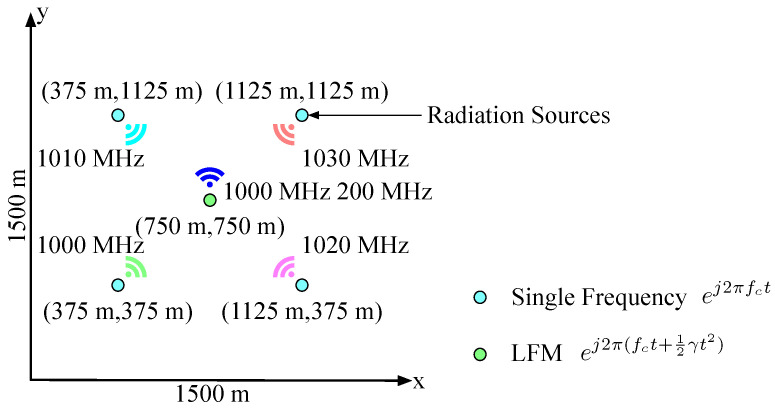
Simulation scene.

The physical space in the simulation is a square region with side length 1500m. Furthermore, there are five radiation sources at points (375,375), (375,1125), (1125,375), (1125,1125), and (750,750), and the first four radiation sources emit single frequency signal $e^{j2\pi f_{c}t}$ with frequencies 1000MHz, 1010MHz, 1020MHz and 1030MHz. The last radiation source emits linear frequency modulation (LFM) signal $e^{j2\pi (f_{c}t+\frac{1}{2}\gamma t^{2})}$ with carrier frequency 1000MHz and bandwidth 200MHz. The above scene is illustrated in Figure 6.

As the radar equation, the power of signal declines approximately as the fourth power of range. Thus, the electromagnetic signal is formulated as

(16) $$S_{r}\left(x,t\right)=\sum_{i=1}^{n_{s}}\frac{S_{i}\left(t-\frac{|x-x_{T}\left|+\right|x_{i}-x_{T}|}{c}\right)}{|x-x_{T}\left|\right|x_{i}-x_{T}|},$$

where $S_{r}\left(x,t\right)$ means the signal on points x at time *t*; $S_{i}\left(t\right)$ and $x_{i}$ means the emission signal and position of the *i*th radiation source, respectively; $x_{T}$ is the position of target; and *c* indicates the velocity of light. The direct propagated signal of radiation source is ignored in Equation (16), as it can be eliminated with direct-path cancellation technique.

There are four receivers located uniformly in the simulated physical space, i.e., the receivers are at positions (500,500),(500,1000),(1000,500), and (1000,1000). Actually, the real detection works in CEMS are generally classified as two sorts, one is passive detection case, in which the information of radiation sources is fully unknown including positions, waveforms and so on, such as passive radar case, and the other is active detection case that some radiation sources are cooperative, which means the information of part of radiation sources such as emission signal is known, but the rest of radiation sources are still unknown.

### 4.2. Passive Detection

In passive detection case, the information of radiation sources is fully unknown including positions, waveforms, etc. Because of the absence of the knowledge concerning signal, the traditional detection method in this situation is the energy detector [38]. In the proposed geometric detector, the little knowledge of the signal leads to preprocessing being unavailable, thus the observed signal would be modeled as a distribution, directly. Because the noise is often Gaussian or asymptotical Gaussian and the central limit theorem is usually satisfied, the observed data are modeled as normal distribution. However, when the length of observed data is considerable, their covariance matrix is too large to operate. Therefore, their correlation length is assumed as $L_{c}$. Suppose that the observed signal is a stationary stochastic process; the mean is estimated as the average of observed signal. Furthermore, the covariance matrix is estimated as

(17) $$\hat{C}=\left[\begin{array}{cccc} c_{0} & c_{1} & \cdots & c_{L_{c}}\\ c_{-1} & c_{0} & \cdots & c_{L_{c}-1}\\ \vdots & \vdots & \ddots & \vdots \\ c_{-L_{c}} & c_{-(L_{c}-1)} & \cdots & c_{0} \end{array}\right],$$

where $c_{k}$ is the correlation sequence as

(18) $$c_{k}=c_{-k}^{*}=\frac{1}{N}\sum_{i=1}^{N}\left(I_{p}\left(t_{i}\right)-\hat{\mu }\right){\left(I_{p}\left(t_{i-k}\right)-\hat{\mu }\right)}^{*},$$

where the superscript * means the conjugate number, $\hat{\mu }$ is the average of observed signal, $I_{p}\left(t\right)$ is the observed signal in position p, $t_{k}$ is the *k*th sampling time and *N* is the number of sampled points. Furthermore, the parameters in initial condition, i.e., the parameters of $H_{0}$ distribution, are $\mu_{H_{0}}=0,C_{H_{0}}=\sigma_{H_{0}}^{2}I$, where $\sigma_{H_{0}}^{2}$ is the power of noise. In [28], the results show the detector using KL divergence instead of Riemannian distance is easier to calculate and has better performance. Therefore, in the simulation, the Riemannian distance between the estimated distribution and $H_{0}$ distribution was calculated by using KL divergence as

(19) $$KL\left(\hat{\mu },\hat{C}∥\mu_{H_{0}},C_{H_{0}}\right)=\frac{1}{2}\left(tr\left(\frac{\hat{C}}{\sigma_{H_{0}}^{2}}-I\right)-\ln|\frac{\hat{C}}{\sigma_{H_{0}}^{2}}|+\frac{{\hat{\mu }}^{H}\hat{\mu }}{\sigma_{H_{0}}^{2}}\right),$$

where $|\hat{C}/\sigma_{H_{0}}^{2}|$ means the determinant of matrix $\hat{C}/\sigma_{H_{0}}^{2}$ and the superscript *H* means the Hermitian transpose.

In passive detection case, energy detector is a traditional detection method for unknown signal. In the simulation, energy detector was compared with geometric detector under same data. As introduced in Section 3, in geometric detection method, the difference between initial CEMS and current CEMS are modeled as a function $\Gamma_{\hat{s},s_{H_{0}}}$, and the norm of the function is the test statistic of geometric detector. Similarly, in energy detector, the difference of two CEMSs is modeled as energy field as Equation (20) and the test statistic is the norm of energy field.

(20) $$E_{p_{k}}=\sum_{i=1}^{N}{\left|I_{p_{k}}\left(t_{i}\right)\right|}^{2}$$

In Equation (20), $p_{k}$ is the position of *k*th receiver, $t_{k}$ is the *k*th sampling time and *N* is the number of sampling points of electromagnetic signal.

The power of electromagnetic signal approximately declines as the fourth power of range (Equation (16)). Therefore, the power of observed signal of the nearest point to the target is the largest and points that are far away from the target might hardly detect the echo from target, tbhs both the function $\Gamma_{\hat{s},s_{H_{0}}}\left(p\right)$ and energy field of the nearest point are supposed to be the largest, meaning the position of the target can be regarded as the same as the position of the nearest point in the sampled $\Gamma_{\hat{s},s_{H_{0}}}\left(p\right)$ and energy field. However, when the power of signal is not large enough, the accuracy of estimated parameter might be extremely low, which means the energy and $\Gamma_{\hat{s},s_{H_{0}}}\left(p\right)$ of the nearest point might not be the highest in all receivers. In general, when the target is more obvious in either $\Gamma_{\hat{s},s_{H_{0}}}\left(p\right)$ or energy field, the detector has better performance.

**Figure 7:**
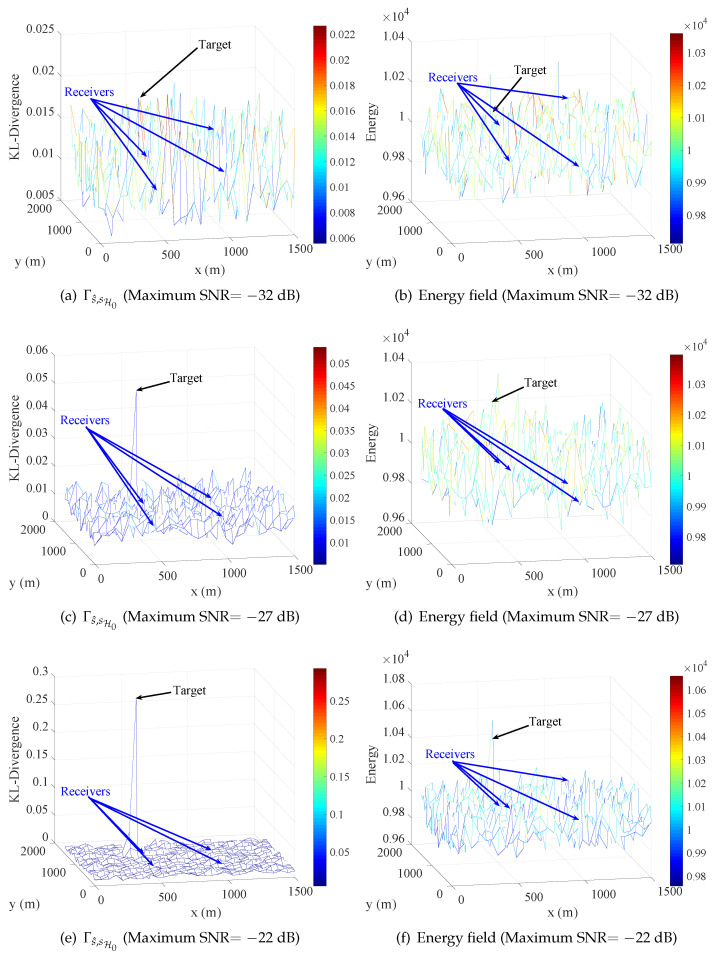
Function $\Gamma_{\hat{s},s_{H_{0}}}$ and Energy field. The modeled difference of initial CEMS and current CEMS in geometric detector and energy detector, respectively.

Let the correlation length $L_{c}=20$ and the sampling frequency be 10GHz, the observed signal is sampled for 1μs, thus the length of observed signal is 10,000. To simplify the related condition, the power of the five radiation sources are equivalent and the power of noise is the same of all four receivers. Furthermore, the noise obeys complex normal distribution that the real part and imaginary part are independent and have the same power. Furthermore, the power of noise was considered a known parameter in the simulation. The radio signal of each receiver was calculated by Equation (16), and SNR of each receiver was determined by the power of the signal and noise. Under the above condition and when the target appears at position (450,1050), the function $\Gamma_{\hat{s},s_{H_{0}}}\left(p\right)$ and energy field under different maximum SNR of the four receivers are shown in Figure 7.

**Figure 8:**
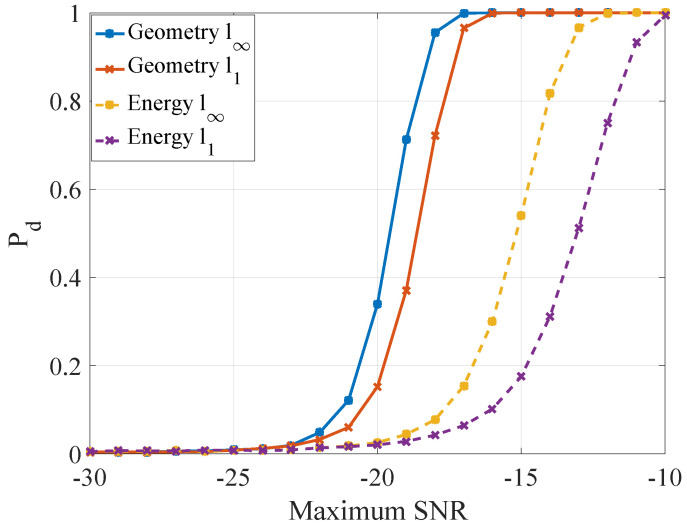
Detection performance curve.

The detection performance curves are shown in Figure 8, which were obtained by $10^{4}$ Monte Carlo runs.

In Figure 7, the results correspond to the above analysis that the target stands out in half of all figures. Figure 7a,b, drawn under maximum SNR =−32dB, shows the target is overwhelmed in due to the low maximum SNR. In Figure 7e,f, maximum SNR =−22dB, the $\Gamma_{\hat{s},s_{H_{0}}}\left(p\right)$ and energy in position of target are relatively large compared to other places, but the target is more prominent in $\Gamma_{\hat{s},s_{H_{0}}}\left(p\right)$. Moreover, it is more obvious that the target stands out in Figure 7c but is overwhelmed in Figure 7d, in which maximum SNR is equivalent to −27dB. Therefore, the relative difference between the nearest point and far point to target is much more obvious in $\Gamma_{\hat{s},s_{H_{0}}}\left(p\right)$ than energy field, which means the target might be easier to detect from noise with the detector using $\Gamma_{\hat{s},s_{H_{0}}}\left(p\right)$ than the detector using energy field. As introduced in Section 3, the geometric detector utilizes the norm of $\Gamma_{\hat{s},s_{H_{0}}}\left(p\right)$ as test statistic, and the norm can also be used in energy field. In the simulation, two relative extreme norms, $l_{1}$ norm and $l_{\infty }$ norm, were considered.

Through changing the power of radiation sources or the power of noise, the maximum SNR can change to every positive number. Under different maximum SNR of the four receivers, we compared the probability of detection of geometric detector and energy detector in two norms. The decision strategies of two detectors are based on Neyman–Pearson criterion. The probability of false-alarm is often equivalent to a relatively small value, and it was considered $P_{F}=5\times 10^{-3}$ in the simulation. The two thresholds of geometric detector were obtained by $10^{5}$ Monte Carlo [39] runs and the $l_{1}$ norm and $l_{\infty }$ norm thresholds of energy detector are formulated as Equations (21) and (22), respectively.

(21) $$\eta_{l_{1}}=\frac{1}{2}Q_{X^{2}\left(1800N\right)}^{-1}\left(P_{F}\right)$$

(22) $$\eta_{l_{\infty }}=\frac{1}{2}Q_{X^{2}\left(2N\right)}^{-1}\left(1-\sqrt[{900}]{1-P_{F}}\right)$$

In Equations (21) and (22), the function $Q_{X^{2}\left(k\right)}^{-1}\left(x\right)$ is the inverse *Q*-function of chi-squared distribution with *k* degrees of freedom, *N* is the length of observed signal of one receiver and $P_{F}$ is the false-alarm probability. Furthermore, the detailed derivation processes of Equations (21) and (22) are in Appendix B.

In Figure 8, with the same norm, the performance of geometric detector is better than energy detector with 4∼5 dB, and the $l_{\infty }$ norm is better than $l_{1}$ norm in same detection method. The reason for the better performance of $l_{\infty }$ norm is that the value of $l_{1}$ norm consists of the entire difference of sequence as Equation (14), but the change of CEMS often occurs in a local area, which would be focused on by $l_{\infty }$ norm as Equation (15). It is worth mentioning that the target stands out in maximum SNR =−27dB in Figure 7 but is not detected in such SNR. The reason is that, when the receivers are not near the target, the $\Gamma_{\hat{s},s_{H_{0}}}$ is not obviously large in the receivers’ locations.

### 4.3. Active Detection

In active detection case, some radiation sources are cooperative in the detection. The information of these radiation sources is known, but the rest of the radiation sources are still unknown. That means the electromagnetic signal in CEMS consists of two parts. One is the desired signal emitted by us, and the other is the interference signal deriving from the unknown radiation sources. In this case, the matched filter (MF) [40] is a commonly used method to detect the emitted signal in the received signal. Actually, in complex electromagnetic environment, the received signal contains not only the emitted signal but also the signals from other radiation sources, which could result in the mismatch with the template signal.

As the known information of cooperative radiation sources, modifying the geometric detection method applied in passive detection case might achieve a better detection performance in this situation. Different from the passive detection case, the observed signal is preprocessed with MF to obtain the SNR gain, because the knowledge concerning some radiation sources is available, thus the processed signal is input into geometric detector as passive detection case.

It should be mentioned that the noise is not white after matched filtering, which means the initial section $s_{0}$ is not the same as in the passive detection case. The correlation sequence of noise is

(23) $$c^{\prime }=\lambda F^{-1}(|F\left(s_{T}\right){|}^{2}),$$

where F means the discrete Fourier transform (DFT), $F^{-1}$ means the inverse DFT, $s_{T}$ indicates the template signal and λ is power normalization factor. Therefore, the covariance matrix of received signal under $H_{0}$ is

(24) $$C_{H_{0}}=\left[\begin{array}{cccc} c_{0}^{\prime } & c_{1}^{\prime } & \cdots & c_{L_{c}}^{\prime }\\ c_{1}^{\prime *} & c_{0}^{\prime } & \cdots & c_{L_{c}-1}^{\prime }\\ \vdots & \vdots & \ddots & \vdots \\ c_{L_{c}}^{\prime *} & c_{L_{c}-1}^{\prime *} & \cdots & c_{0}^{\prime } \end{array}\right],$$

where $c_{k}^{\prime }$ indicates the *k*th element of $c^{\prime }$ and superscript ∗ means the conjugate number. Equation (19) would transform to the standard form

(25) $$KL\left(\hat{\mu },\hat{C}∥\mu_{H_{0}},C_{H_{0}}\right)=\frac{1}{2}\left(tr\left(\hat{C}C_{H_{0}}^{-1}-I\right)-\ln|\hat{C}C_{H_{0}}^{-1}|+{\hat{\mu }}^{H}C_{H_{0}}^{-1}\hat{\mu }\right).$$

**Figure 9:**
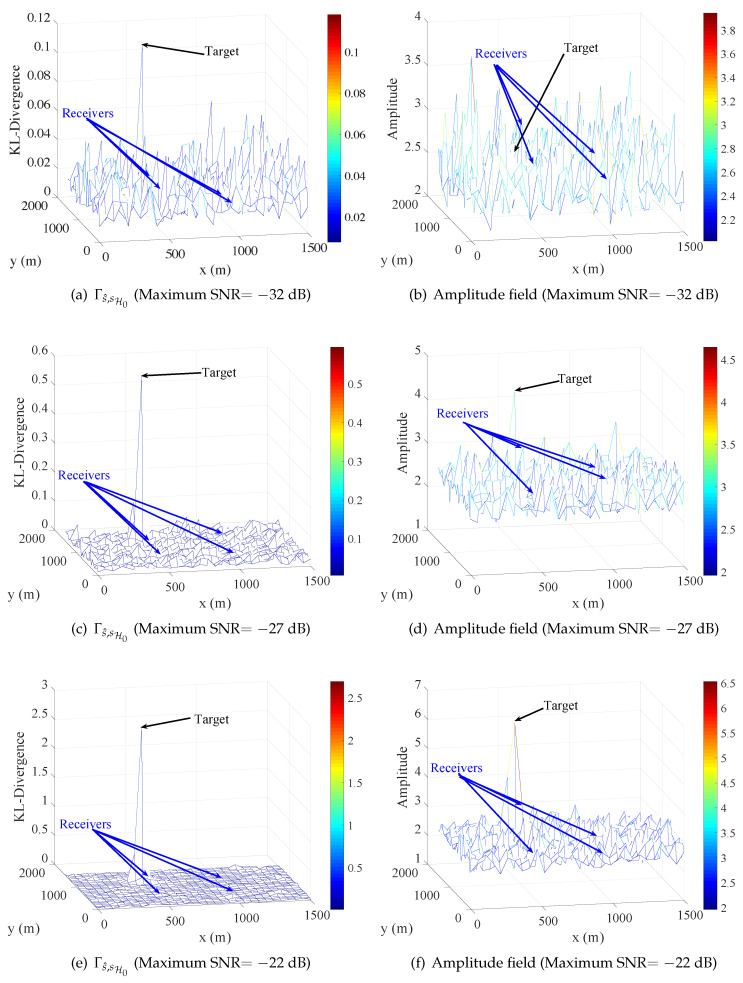
Function $\Gamma_{\hat{s},s_{H_{0}}}$ and matched filter amplitude field. The modeled difference of initial CEMS and current CEMS in geometric detector and matched filter, respectively.

In active detection simulation, the radiation source at (750,750), which emits LFM signal, is supposed to be cooperative in the detection, which means the emission signal of this source can be utilized as template signal in MF. The other conditions, such as target and sampling frequency, are as same as passive detection scene. The other method for comparison is based on MF, in which the difference between initial CEMS and current CEMS is modeled as the maximum amplitude among the processed signal by MF. The function $\Gamma_{\hat{s},s_{H_{0}}}\left(p\right)$ and maximal amplitude field of matched filtered signal is shown in Figure 9. The target stands out in function $\Gamma_{\hat{s},s_{H_{0}}}\left(p\right)$ even though maximum SNR =−32dB, and the target is overwhelmed in passive detection case under same condition. In amplitude field, target stands out under maximum SNR =−27dB and maximum SNR =−22dB but is overwhelmed under maximum SNR =−32dB, which is much better than energy field in passive detection case.

**Figure 10:**
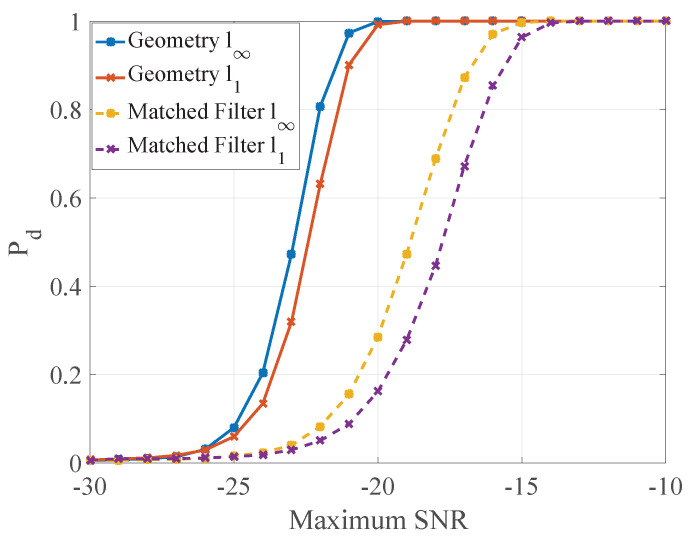
Detection performance curve.

Similarly, based on NP criterion and $P_{F}=5\times 10^{-3}$, we compared the probability of detection of geometric detector and matched filter in two norms. The two thresholds of geometric detector were also obtained by $10^{5}$ Monte Carlo runs. Furthermore, the thresholds of $l_{1}$ norm and $l_{\infty }$ norm of matched filter might be hard to work out the analytical expressions due to the color noise, thus they were also obtained by $10^{5}$ Monte Carlo runs. The detection performance curves are shown in Figure 10; the four curves were obtained by $10^{4}$ Monte Carlo runs. With the same norm, the performance of geometric detector is better than matched filter with 4∼5 dB. Because the knowledge of the four other radiation sources is absent, the echo of object mismatch the template signal of MF, which results in the unexpected performance of detection. In geometric detector, the test statistic derives from the difference of probability distribution, and the mismatch of MF would not disturb this character. Therefore, the performance of geometric is better than MF in this situation. As in the passive detection case, the performance of different norm correspond to the analysis of passive detection case: $l_{\infty }$ norm also outperforms $l_{1}$ norm approximately 4∼5 dB.

## 5. Conclusions

In this paper, the complex electromagnetic space (CEMS) is modeled as a section of vector bundle that consists of statistical manifold and physical space. Then, the vital point of change detection in CEMS is quantifying the difference of sections. Based on information geometry, the distance between two sections is defined with Riemannian metric and the norm of function and a geometric detection method is proposed with the section distance. In simulation, the performance of the proposed geometric detector is compared with energy detector and matched filter detection method in passive case and active case, respectively. The results show the geometric detector achieves better performance than both energy detector and matched filter, with 4∼5 dB improvements of SNR, in the two scenes.

## Appendix A. The proof of Theorem 1

**Proof.** Positive definiteness.

(A1) $$\begin{array}{cc} D_{s}\left(s_{1},s_{2}\right) & =∥\Gamma_{s_{1},s_{2}}{∥}_{k}\\ & =(\int_{N}|\;\Gamma_{s_{1},s_{2}}\left(p\right)\;{{|}^{k}\;dp)}^{\frac{1}{k}}\\ & =(\int_{N}|\;D_{M}\left(\pi_{M}\left(s_{1}\left(p\right)\right),\pi_{M}\left(s_{2}\left(p\right)\right)\right)\;{{|}^{k}\;dp)}^{\frac{1}{k}}\geq 0 \end{array}$$

Because the function $\Gamma_{s_{1},s_{2}}\left(p\right)$ is continuous, Equation (A1) equals to 0, if and only if $\Gamma_{s_{1},s_{2}}\left(p\right)\equiv 0$, which means $D_{M}\left(\pi_{M}\left(s_{1}\left(p\right)\right),\pi_{M}\left(s_{2}\left(p\right)\right)\right)\equiv 0$. Because $D_{M}$ is the distance on statistical manifold, $D_{M}\left(\pi_{M}\left(s_{1}\left(p\right)\right),\pi_{M}\left(s_{2}\left(p\right)\right)\right)\equiv 0$ means that $s_{1}\left(p\right)\equiv s_{2}\left(p\right)$ for each p, i.e., $s_{1}=s_{2}$. Symmetry.

(A2) $$\begin{array}{cc} D_{s}\left(s_{1},s_{2}\right) & =∥\Gamma_{s_{1},s_{2}}{∥}_{k}\\ & =(\int_{N}|\;\Gamma_{s_{1},s_{2}}\left(p\right)\;{{|}^{k}\;dp)}^{\frac{1}{k}}\\ & =(\int_{N}|\;D_{M}\left(\pi_{M}\left(s_{1}\left(p\right)\right),\pi_{M}\left(s_{2}\left(p\right)\right)\right)\;{{|}^{k}\;dp)}^{\frac{1}{k}} \end{array}$$

Because $D_{M}$ is the distance on statistical manifold, it satisfies the symmetry, and we can get

(A3) $$\begin{array}{cc} D_{s}\left(s_{1},s_{2}\right) & =(\int_{N}|\;D_{M}\left(\pi_{M}\left(s_{1}\left(p\right)\right),\pi_{M}\left(s_{2}\left(p\right)\right)\right)\;{{|}^{k}\;dp)}^{\frac{1}{k}}\\ & =(\int_{N}|\;D_{M}\left(\pi_{M}\left(s_{2}\left(p\right)\right),\pi_{M}\left(s_{1}\left(p\right)\right)\right)\;{{|}^{k}\;dp)}^{\frac{1}{k}}\\ & =(\int_{N}|\;\Gamma_{s_{2},s_{1}}\left(p\right)\;{{|}^{k}\;dp)}^{\frac{1}{k}}\\ & =D_{s}\left(s_{2},s_{1}\right) \end{array}$$

It means $D_{s}\left(s_{1},s_{2}\right)$ satisfies the symmetry. Triangle inequality. At first, we should show that, for each pair non-negative function $f_{1},f_{2}$, the $L_{k}$-norms of them satisfy

(A4) $$∥f_{1}+f_{2}{∥}_{k}\geq ∥f_{1}{∥}_{k}$$

It is obvious because of

(A5) $$\begin{array}{cc} ∥f_{1}+f_{2}{∥}_{k} & =(\int |\;f_{1}\left(x\right)+f_{2}\left(x\right)\;{{|}^{k}\;dx)}^{\frac{1}{k}}\\ & \geq (\int |\;f_{1}\left(x\right)\;{{|}^{k}\;dx)}^{\frac{1}{k}}=∥f_{1}{∥}_{k} \end{array}$$

Because $D_{M}$ satisfies triangle inequality,

(A6) $$\begin{array}{c} \Gamma_{s_{1},s_{3}}+\Gamma_{s_{3},s_{2}}-\Gamma_{s_{1},s_{2}}\\ =D_{M}\left(\pi_{M}\left(s_{1}\left(p\right)\right),\pi_{M}\left(s_{3}\left(p\right)\right)\right)+D_{M}\left(\pi_{M}\left(s_{3}\left(p\right)\right),\pi_{M}\left(s_{2}\left(p\right)\right)\right)-D_{M}\left(\pi_{M}\left(s_{1}\left(p\right)\right),\pi_{M}\left(s_{2}\left(p\right)\right)\right)\\ \geq 0 \end{array}$$

Then, because of Equation (A5),

(A7) $$\begin{array}{cc} D_{s}\left(s_{1},s_{2}\right) & =∥\Gamma_{s_{1},s_{2}}{∥}_{k}\\ & \leq ∥\Gamma_{s_{1},s_{2}}+\left(\Gamma_{s_{1},s_{3}}+\Gamma_{s_{3},s_{2}}-\Gamma_{s_{1},s_{2}}\right){∥}_{k}=∥\Gamma_{s_{1},s_{3}}+\Gamma_{s_{3},s_{2}}{∥}_{k}\\ & \leq ∥\Gamma_{s_{1},s_{3}}{∥}_{k}+∥\Gamma_{s_{3},s_{2}}{∥}_{k}=D_{s}\left(s_{1},s_{3}\right)+D_{s}\left(s_{3},s_{2}\right) \end{array}$$

Overall, $D_{s}$ satisfies the positive definiteness, symmetry and triangle inequality, if the norm of function is $L_{k}$-norm. □

## Appendix B. The Derivation Process of Equations (21) and (22)

Under $H_{0}$, the signal $I_{p_{k}}\left(t_{i}\right)$ obeys complex normal distribution with zero mean and variance 1, so the $l_{1}$ norm of energy field is

(A8) $$∥E{∥}_{1}=\sum_{k=1}^{900}E_{p_{k}}=\sum_{k=1}^{900}\sum_{i=1}^{N}|w_{p_{k}}\left(t_{i}\right){|}^{2}=\sum_{k=1}^{900}\sum_{i=1}^{N}|w_{p_{k}}^{\left(r\right)}\left(t_{i}\right){|}^{2}+{\left|w_{p_{k}}^{\left(i\right)}\left(t_{i}\right)\right|}^{2},$$

where $w_{p_{k}}^{\left(r\right)}\left(t\right)$ is the real part of $w_{p_{k}}\left(t\right)$ and $w_{p_{k}}^{\left(i\right)}\left(t\right)$ is the imaginary part, and both $w_{p_{k}}^{\left(r\right)}\left(t\right)$ and $w_{p_{k}}^{\left(i\right)}\left(t\right)$ obey the normal distribution with zero mean and variance $\frac{1}{2}$. That means $2∥E{∥}_{1}∼X^{2}\left(1800N\right)$, thus the threshold equals to the right side of Equation (21).

Similarly, the $l_{\infty }$ norm of energy field is

(A9) $$∥E{∥}_{\infty }=\max_{k=1,\ldots ,900}E_{p_{k}}=\max_{k=1,\ldots ,900}\sum_{i=1}^{N}|w_{p_{k}}^{\left(r\right)}\left(t_{i}\right){|}^{2}+{\left|w_{p_{k}}^{\left(i\right)}\left(t_{i}\right)\right|}^{2}.$$

Furthermore, according to the statistical theory [41],

(A10) $$P\left(∥E{∥}_{\infty }\geq \eta_{l_{\infty }}\right)=1-\prod_{k=1}^{900}\left(1-P\left(E_{p_{k}}\geq \eta_{l_{\infty }}\right)\right),$$

where *P* means the probability. Because all of $2E_{p_{k}}\;\left(k=1,\ldots ,900\right)$ obey same distribution $X^{2}\left(2N\right)$, Equation (A10) can be expressed as

(A11) $$P\left(∥E{∥}_{\infty }\geq \eta_{l_{\infty }}\right)=1-{\left(1-Q_{X^{2}\left(2N\right)}\left(2\eta_{l_{\infty }}\right)\right)}^{900}.$$

Let $P\left(∥E{∥}_{\infty }\geq \eta_{l_{\infty }}\right)=P_{F}$; we can get Equation (22).
